# A case of Rowell syndrome after a herpes simplex infection and prolonged sun exposure

**DOI:** 10.1002/ski2.378

**Published:** 2024-04-01

**Authors:** Ariana Palacio, Karla Medrano Cebrian, Michael Majores, Manfred Uerlich, Erhard Bierhoff, Sonja Bonness, Uwe Reinhold

**Affiliations:** ^1^ MVZ Dermatologisches Zentrum Bonn Bonn Germany; ^2^ Heinz‐Werner‐Seifert Institut für Dermatopathologie Bonn Germany

## Abstract

Rowell Syndrome is a rare entity combining erythema exudativum multiforme (EEM) and lupus erythematosus (LE). Zeitouni et al. redefined Rowell Syndrome's diagnostic criteria. Major criteria include: (1) LE (systemic, discoid or subacute cutaneous), (2) EEM‐like skin lesions and (3) speckled pattern of antinuclear antibodies. Minor criteria comprise: (1) chilblains, (2) positive anti‐SSA/Ro or anti‐SSB/La antibodies and (3) positive rheumatoid factor. The diagnosis is achieved when all major criteria and at least one minor criterion are present. Prognosis and treatment regimens are those of EEM and LE, with reported good response to oral cortisone, azathioprine, cyclosporine, dapsone, antimalarials and methotrexate. We present a case of Rowell Syndrome in a young adult after a herpes simplex type 1 infection and unprotected sun exposure, with good response to both topical corticosteroids and calcineurin‐inhibitors.

## Graphical abstract

A 43‐year‐old male with previous history of alopecia areata presented with an erythema exudativum multiforme (EEM) after a herpes simplex type 1 infection, as well as subacute cutaneous lupus erythematosus (SCLE) lesions after severe sun exposure during a trip to the Dominican Republic. The combination of EEM and SCLE is a rare condition known as Rowell Syndrome. Our patient responded well to topical corticosteroids and calcineurin inhibitors.

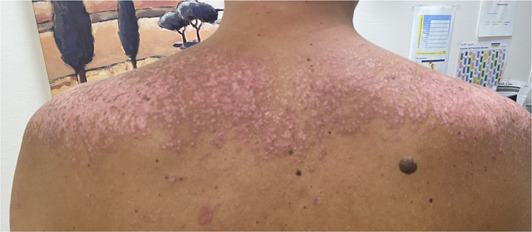

the image chosen for the graphical abstarct is not the most representative. If an image needs to be added here, I would advise to use one of the arms or back.

## 1 INTRODUCTION

We present a case of an otherwise healthy 43‐year‐old man of Balkan descent with a 3‐week history of general malaise and skin rashes on his trunk and upper extremities. The symptoms appeared at the beginning of his holidays in the Dominican Republic after the first day of sun exposure without sunscreen and subsequent sunburn on his neck and shoulders. The patient reported no intake of medication and did not have any known illnesses other than an unclear relapsing alopecia in stress phases with good response to topical minoxidil and betamethasone.

**FIGURE 1:**
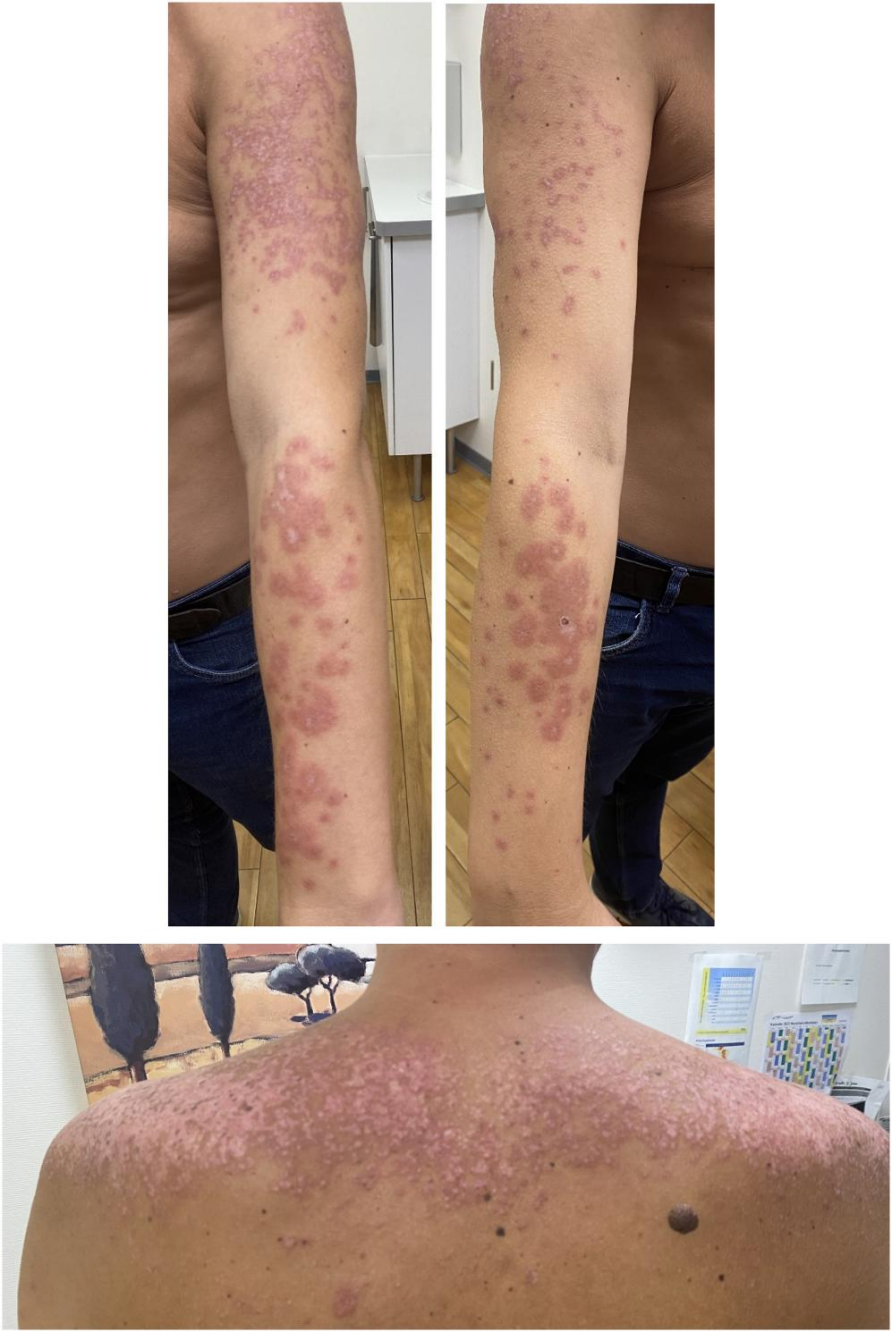
Annular plaques with a polycyclic pattern and erythematous to violet elevated borders and central pallor on both upper extremities (above) and on the back (below).

During the physical examination, annular plaques with erythematous to violet elevated borders and central pallor were identified on the dorsal side of both upper extremities and on the upper third of the patient's back. Some of the plaques merged creating a polycyclic pattern, mainly on both forearms, left upper arm and back (see Figure 1). The patient described having experienced the same skin changes the year before in the Dominican Republic, which had a self‐limiting course with completed healing after some weeks. These clinical findings correlated well with a subacute cutaneous lupus erythematosus (SCLE), probably triggered by solar exposure.

**FIGURE 2:**
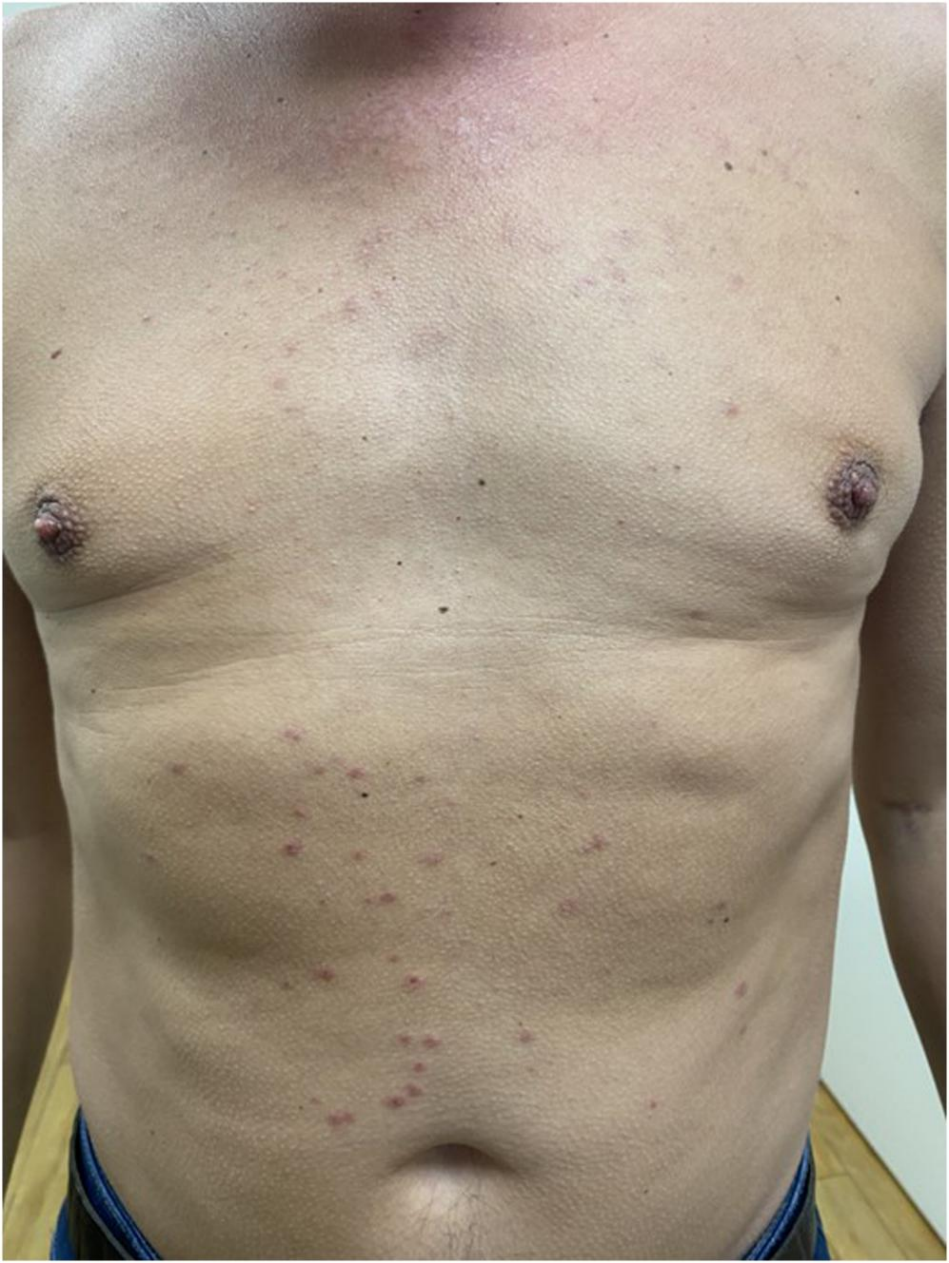
Symmetric erythematous cocarde‐like papules with central blistering on the abdominal area.

Additionally, 1–4 mm erythematous cocarde‐like papules with central blistering were found on the patient's chest and middle abdominal area, extending symmetrically to both sides (see Figure 2). The patient also reported having had those skin efflorescences on both palms and soles, as well as on the bearded areas of his cheeks during his vacation. A 3 mm × 4 mm scab was located on the lateral side of the lower lip. According to the patient, small vesicles and afterwards an ulcer appeared on the area at the beginning of his trip. This constellation supports the diagnosis of an erythema exudativum multiforme (EEM) induced by a herpes simplex virus infection.

Three well‐defined patches with diffuse thinning of the hair were located on the parieto‐occipital area of the scalp. The patient remembered noticing these plaques about 1 month ago. Due to previous medical history and good response to topical corticosteroids, this relapsing stress‐associated hair loss was compatible with alopecia areata.

Blood tests showed leucopenia, elevated liver enzymes, elevated C‐reactive protein (CRP), positive rheumatoid factor, high titres of speckled pattern antinuclear antibodies (ANA) (1:2.560) and positive anti‐SSA/Ro, anti‐SSB/La, anti‐U1‐RNP and antiphospholipid antibodies (lupus anticoagulant, anti‐cardiolipin antibody and anti‐β2‐glycoprotein‐I antibody). Anti‐dsDNA and anti‐Sm were negative. Regarding serologic testing, IgG antibodies against HSV‐1 were detected.

**FIGURE 3:**
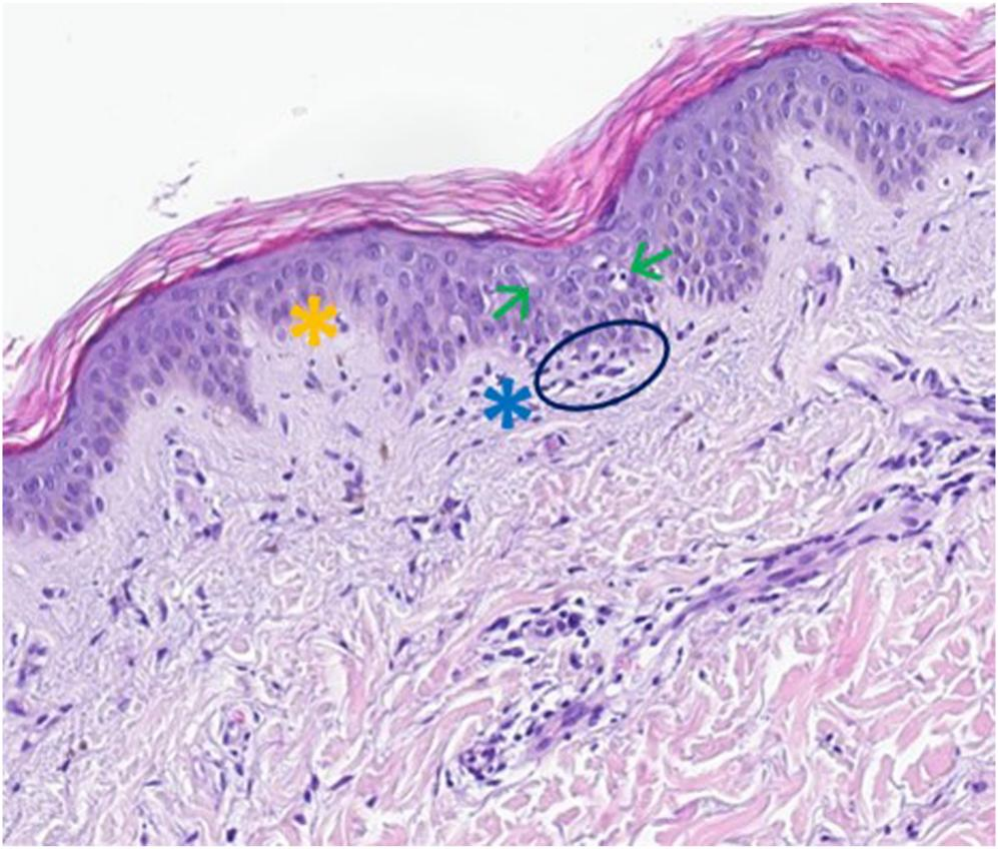
Skin biopsy—histology of erythema exudativum multiforme. Vacuolisation of the epidermis (green arrows), destruction of the basal membrane (yellow asterisk), lichenoid inflammation with lymphocytic infiltrate (blue asterisk) and apoptotic keratinocytes (blue circle).

**FIGURE 4:**
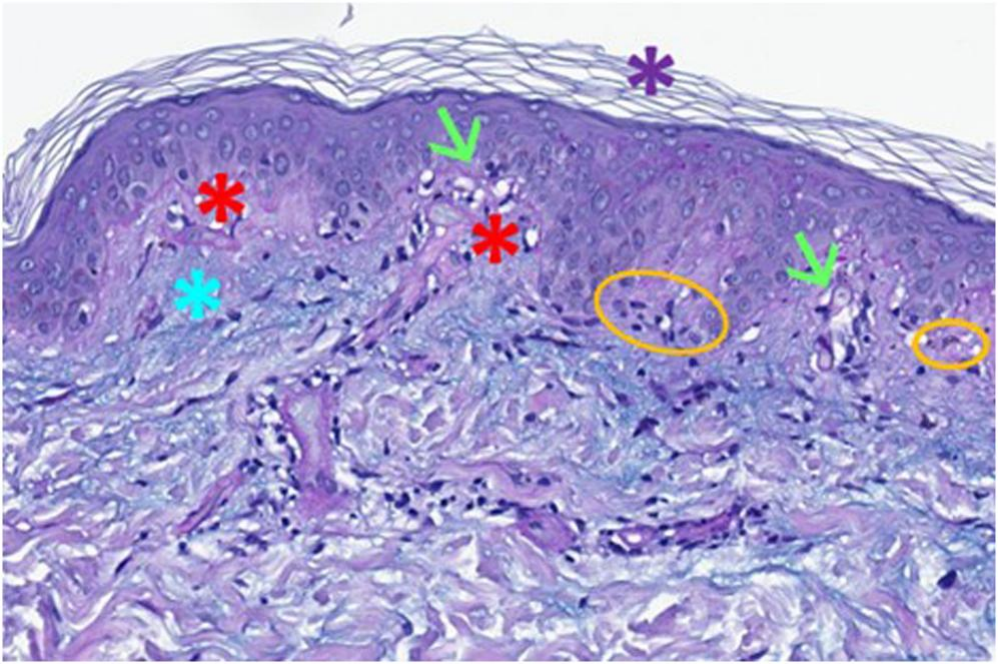
Skin biopsy—histology of subacute cutaneous lupus erythematosus. Hyperorthokeratosis (purple asterisk), interface dermatitis with dense lymphocytic infiltration (red asteriscs), vacuolar degeneration of the basal layer (green arrows), dyskeratotic keratinocytes (orange circles), subcutaneous mucine deposition (blue asterisk).

Skin biopsies of two affected areas (abdominal area and right posterior forearm) displayed characteristic histological findings of erythema multiforme (vacuolar degeneration of the epidermis with destruction of the basal membrane, lichenoid inflammation with dense lymphocytic infiltrate and apoptotic keratinocytes; see Figure 3) and subacute cutaneous lupus erythematosus (hyperorthokeratosis, interface‐dermatitis with vacuolisation of the basal layer of the epidermis, dyskeratotic keratinocytes, dense lymphotic infiltrate and mucin deposition within connective tissue; see Figure 4), confirming the diagnosis of a Rowell Syndrome.

Our patient was initially treated symptomatically with topical triamcinolone acetonide 0.1% (twice daily for 1 week, once daily for 1 week) followed by topical pimecrolimus (once daily for 2 weeks). A combination of topical minoxidil and betamethasone was applied on the alopecia patches on the scalp. The use of sunscreen was advised. After completing the treatment, all skin lesions were healed without scarring. A slight hypopigmentation was identified on the forearms. Two months later, early signs of hair regrowth were visible on the alopecic plaques without signs of scarring. A new blood test 4 weeks after the first consultation showed a correction of the leucopenia, a reduction of CRP and a reduction of the liver enzymes.

A score of 15 on the 2019 EULAR/ACR classification criteria for systemic lupus erythematosus (SLE) was achieved, increasing the likelihood of a SLE. The patient was referred to his family doctor for an hepatic ultrasound and monitoring of cardiovascular factors. A participation in general screening programs was recommended, including skin cancer screening, colorectal cancer screening and PSA‐test. Further examinations were conducted by the rheumatologists, due to joint pain described by the patient.

Rowell Syndrome^1^ is a rare entity combining EEM and lupus erythematosus (LE).^2^, ^3^, ^4^, ^5^, ^6^, ^7^, ^8^ Zeitouni et al. redefined Rowell Syndrome's diagnostic criteria.^2^ Major criteria include: (1) LE (systemic, discoid or subacute cutaneous), (2) EEM‐like skin lesions and (3) speckled pattern of ANA antibodies. Minor criteria comprise: (1) chilblains, (2) positive anti‐SSA/Ro or anti‐SSB/La antibodies and (3) positive rheumatoid factor. The diagnosis is achieved when all major criteria and at least one minor criterion are present.^2^, ^3^, ^4^, ^5^, ^6^, ^7^, ^8^ Prognosis and treatment regimens are those of EEM and LE,^8^ with reported good response to oral cortisone, azathioprine, cyclosporine, dapsone, antimalarials^8^ and methotrexate.^9^

## CONFLICT OF INTEREST STATEMENT

None to declare.

## AUTHOR CONTRIBUTIONS

**Ariana Palacio**: Conceptualization (lead); data curation (lead); formal analysis (lead); investigation (lead); methodology (lead); project administration (lead); writing – original draft (lead); writing – review & editing (lead). **Karla Medrano Cebrian**: Validation (supporting). **Michael Majores**: Formal analysis (supporting). **Manfred Uerlich**: Formal analysis (supporting). **Erhard Bierhoff**: Formal analysis (supporting). **Sonja Bonness**: Validation (supporting). **Uwe Reinhold**: Validation (supporting).

## ETHICS STATEMENT

The patients in this manuscript have given written informed consent to publication of their case details.

## Data Availability

The data underlying this article will be shared on reasonable request to the corresponding author.
